# Novel β-lactam antibiotics versus other antibiotics for treatment of complicated urinary tract infections: a systematic review and meta-analysis

**DOI:** 10.3389/fphar.2024.1420170

**Published:** 2024-10-10

**Authors:** Xiang hua Quan, Xin yi Wang, Chun hua Han, Xiao min Xing, Bin Zhang, Huai qin Cang

**Affiliations:** ^1^ Department of Pharmacy, Affiliated Hospital of Qingdao University, Qingdao, China; ^2^ Department of Clinical Laboratory, Affiliated Hospital of Qingdao University, Qingdao, China

**Keywords:** novel, β-lactam antibiotics, complicated urinary tract infections, systematic review, meta-analysis

## Abstract

**Background:**

Novel β-lactam antibiotics as well as other kinds of antibiotics have been used to treat complicated urinary tract infections (cUTIs); however, their efficacy and safety remain controversial.

**Objective:**

We conducted a systematic review with meta-analysis to explore the efficacy and safety of novel β-lactam antibiotics versus other antibiotics against cUTIs.

**Methods:**

PubMed, Embase, and the Cochrane Central Register of Controlled Trials were searched systematically from inception through 15 March 2024 for clinical trials comparing novel β-lactam antibiotics with other antibiotics for treatment of cUTIs. Random-effects models were used to evaluate the impact of treatment on the risk ratio (RR) of clinical response, microbiologic response, adverse effects (AEs), serious adverse effects (SAEs). The quality of evidence was evaluated with the Cochrane Risk of Bias assessment tool. The review was registered in INPLASY (INPLASY202440054).

**Results:**

Ten randomized controlled trials involving 5, 925 patients met our inclusion criteria. Our meta-analysis revealed that there was no significant difference in overall clinical response (RR = 1.02), AEs (RR = 1.07), SAEs (RR = 1.20) between novel β-lactam antibiotics groups and other antibiotics groups. However, a significant difference was found in a subgroup of clinical cure rates at the end of treatment between novel β-lactam antibiotics groups and carbapenems groups, with low heterogeneity (RR = 1.02). A significant difference was observed in microbiologic response (RR = 1.11). Subgroup analysis revealed a significant difference in microbiologic response between novel BBL/BLS groups and carbapenems groups (RR = 1.13, I^2^ = 21%, P = 0.005). Differences was observed between novel BBL/BLS groups and piperacillin/tazobactam sodium groups (RR = 1.21, I^2^ = 70%, P = 0.02). Similar results were obtained from subgroup analysis of the difference in microbiologic response between novel β-lactam antibiotics groups and ertapenem groups (RR = 0.92, I^2^ = 0, P = 0.01).

**Conclusion:**

Novel β-lactam antibiotics had similar overall clinical cure, AEs, SAE, to other antibiotics in the treatment of cUTIs. However, novel β-lactam antibiotics demonstrated superior clinical cure rates compared to carbapenems in a subgroup analysis, and exhibited better microbiologic response than other antibiotics.

## 1 Introduction

The complicated urinary tract infections (cUTIs), being the second most prevalent infectious disease, were widely recognized as a major burden for healthcare systems (Vallejo-Torres et al., 2018a; Petrosillo et al., 2019; Vallejo-Torres et al., 2018b). cUTIs, which frequently lead to hospitalization and are a common complication during hospital stays, exhibit a higher prevalence of antimicrobial resistance compared with uncomplicated UTIs. In the United States, there were over two million emergency department visits for cUTIs between 2016 and 2018, with approximately two-thirds of these cases requiring hospital admission (Zilberberg et al., 2022a). Furthermore, nearly 700,000 cases were caused by cUTIs, comprising approximately 20% of all annual admissions in 2018. The annual cost of UTIs for governments worldwide amounts to tens of millions of dollars. Due to the dynamic nature of antimicrobial susceptibilities, the availability of timely and appropriate empiric antibiotic coverage plays a pivotal role in mitigating infection-related morbidity and costs. However, shifting antimicrobial susceptibilities have limited treatment options, increasing the likelihood of patients receiving inappropriate empiric therapy despite clinicians’ best intentions (Petrosillo et al., 2019). Several studies have indicated that the hospitalization costs of treating carbapenem-resistant urinary tract infections exceeds $20,000, and this expense further escalates in cases of nosocomial infections (Petrosillo et al., 2019; Zilberberg et al., 2022b).

Gram-negative bacilli, especially uropathogenic *Escherichia coli* (UPEC), are major pathogens implicated in cUTIs (Öztürk and Murt, 2020; Wagenlehner et al., 2020). Other Enterobacteriaceae, including uropathogenic *Klebsiella* spp, *Proteus* spp, and *Pseudomonas aeruginosa* can also cause UTIs (Raman et al., 2018). UPEC is accountable 50% of hospital-acquired (nosocomial) cUTIs. The occurrence cUTIs is commonly observed among the elderly population and individuals with catheterization. The domination of UPEC cells over the host’s urothelial cells compromises the integrity of the host cell’s innate immune system, thereby facilitating chronic colonization and biofilm formation by UPEC pathotypes within the urinary tract cells and tissues. Consequently, this can give rise to severe complications including bacteremia, septicemia, uresepsis, and even mortality in infected patients (Wagenlehner et al., 2020; Zhou et al., 2023; Asadi Karam et al., 2019). Owing to the rapid emergence and dissemination of antimicrobial resistance, including the involvement of extended-spectrum β-lactamase (ESBL)-producing and multidrug-resistant organisms (MDROs), limited therapeutic options are available for some patients with cUTIs. The emergence of antibiotic-resistant strains can be attributed to the inappropriate use and excessive administration of antibiotics, which subsequently results in treatment failures. The incidence of recurrent UTIs within 4–6 months is observed in approximately 20%–30% of women who have previously experienced an initial UTIs. The high recurrence rates of cUTIs and the increasing antimicrobial resistance among uropathogens impose a significant economic burden. Consequently, the inefficacy of antibiotic therapies underscores the urgent necessity for the development of alternative treatment to combat cUTIs (Wagenlehner et al., 2020; Kot, 2019; Sarshar et al., 2020). Several novel β-lactam antibiotics were approved drugs for clinical trials after 2012, such as cefiderocol, sulopenem, and tebipenem pivoxil hydrobromide, novel β-lactam and β-lactamase inhibitors including ceftiazidime/avibactam, cetolozane/tazobactam, meropenem/vaborbactam, imipenem/cialstatin/relebactam, cefeime/tazobactam, ceftaroline/avibactam, cefepime-enmetazobactam, have been used to treat infections caused by MDROs, including cUTIs.

There is limited evidence on whether novel β-lactam antibiotics are superior to other antibiotics in terms of efficacy and safety. This systematic review and meta-analysis was conducted to explore the efficacy and safety of novel β-lactam antibiotics for the treatment of patients with cUTIs.

## 2 Methods

This systematic review and meta-analysis was carried out in accordance with the Preferred Reporting Items for Systematic Reviews and Meta-analyses (PRISMA) reporting guidelines (Page et al., 2021). The meta-analysis protocol was registered in INPLASY (INPLASY202440054).

### 2.1 Search strategy

The database searched were PubMed, Embase, and the Cochrane Central Register of Controlled Trials (CENTRAL) independently, from inception until 15 March 2024, for studies on the efficacy and safety of novel β-lactam antibiotic for treatment of patients with cUTIs. The following search terms were used: “urinary tract infection*,” “UTI,” “bacteriuria,” “pyuria,” “pyelonephritis,” “ceftazidime/avibactam,” “ceftolozane/tazobactam,” “meropenem/vaborbactam,” “imipenem/cilastatin/relebactam,” “cefepime/tazobactam,” “ceftaroline/avibactam,” “cefepime/zidebactam,” “cefepime-enmetazobactam,” “meropenem/nacubactam,” “cefiderocol,” “sulopenem,” and “tebipenem pivoxil hydrobromide.” The outcomes of the search are shown in the Supplementary Material.

### 2.2 Selection criteria

The selection criteria for articles were as follows:

(1) Participants: adult patients diagnosed with cUTI.
(2) Intervention: The experimental group was treated with novel β-lactam antibiotics.
(3) Comparators: The control group was treated with other antibiotics.
(4) Outcome: clinical response, microbiological response, adverse effect, serious adverse effect.
(5) Study design: randomized controlled trials (RCTs).

### 2.3 Exclusion criteria

The following kinds of studies were excluded from the present meta-analysis and systematic review: 1) Abstracts, conference papers; 2) studies with incomplete data or those in which the experimental group was treated with non-novel antibiotics; 3) protocols; 4) *post hoc* analysis; and 5) studies reporting mixed infection.

### 2.4 Study selection and data extraction

Two reviewers independently screened the titles, abstracts, and full text of the articles to identify potentially eligible studies. Disagreements were resolved through consultation with a third reviewer. The following data were extracted from selected studies: author names, year of publication; the country where the study was conducted; study design; population characteristics, number of participants; interventions; drug dose; comparisons.

### 2.5 Assessment of risk of bias

Two reviewers independently evaluated the quality of the included studies using the Cochrane Risk of Bias 2.0 assessment tool (Higgins, 2019). Studies were evaluated as low risk, unclear risk, or high risk based on the following characteristics: random sequence generation (selection bias); allocation concealment (selection bias); blinding of participants, personnel (performance bias); incomplete outcome data (attrition bias); selective reporting (reporting bias); and other bias.

### 2.6 Outcomes

The primary outcome was the overall clinicalresponse, the clinical cure was defined as the complete resolution or substantial improvement of signs and symptoms associated with the index infection, to such an extent that no further antibacterial therapy is required; the secondary outcomes were the clinical cure rate at test of cure (TOC), the clinical cure rate at the end of treatment (EOT), microbiological response, adverse effects (AEs), serious adverse effects (SAEs). Subgroup analyses were performed according to antibiotic type in clinical response at TOC, infection type in clinical response at TOC, microbial pathogens, and common AEs.

### 2.7 Statistical analysis

Relative risk (RR) and 95% confidence intervals (CI) were calculated for dichotomous variables with the DerSimonian–Laird random effects model. Cochran’s Q statistic (significance level, P < 0.01) and I^2^ statistic were determined to evaluate heterogeneity. I^2^ values were used as an approximate guide for categorization, as follows: 0%–40% was considered to indicate low heterogeneity; more than 50%–70% was considered to represent moderate heterogeneity; and more than 70% to represent high heterogeneity. Publication bias was assessed using funnel plots. Subgroup analyses were performed to explore heterogeneity according to antibiotic type, infection type in terms of clinical response, and antibiotic type in terms of microbial pathogens eradiation. Sensitivity analysis was undertaken to ascertain the results of the meta-analysis by excluding each individual study. Review Manager 5.4 was used for this meta-analysis (Higgins, 2019).

## 3 Results

### 3.1 Search results

**FIGURE 1:**
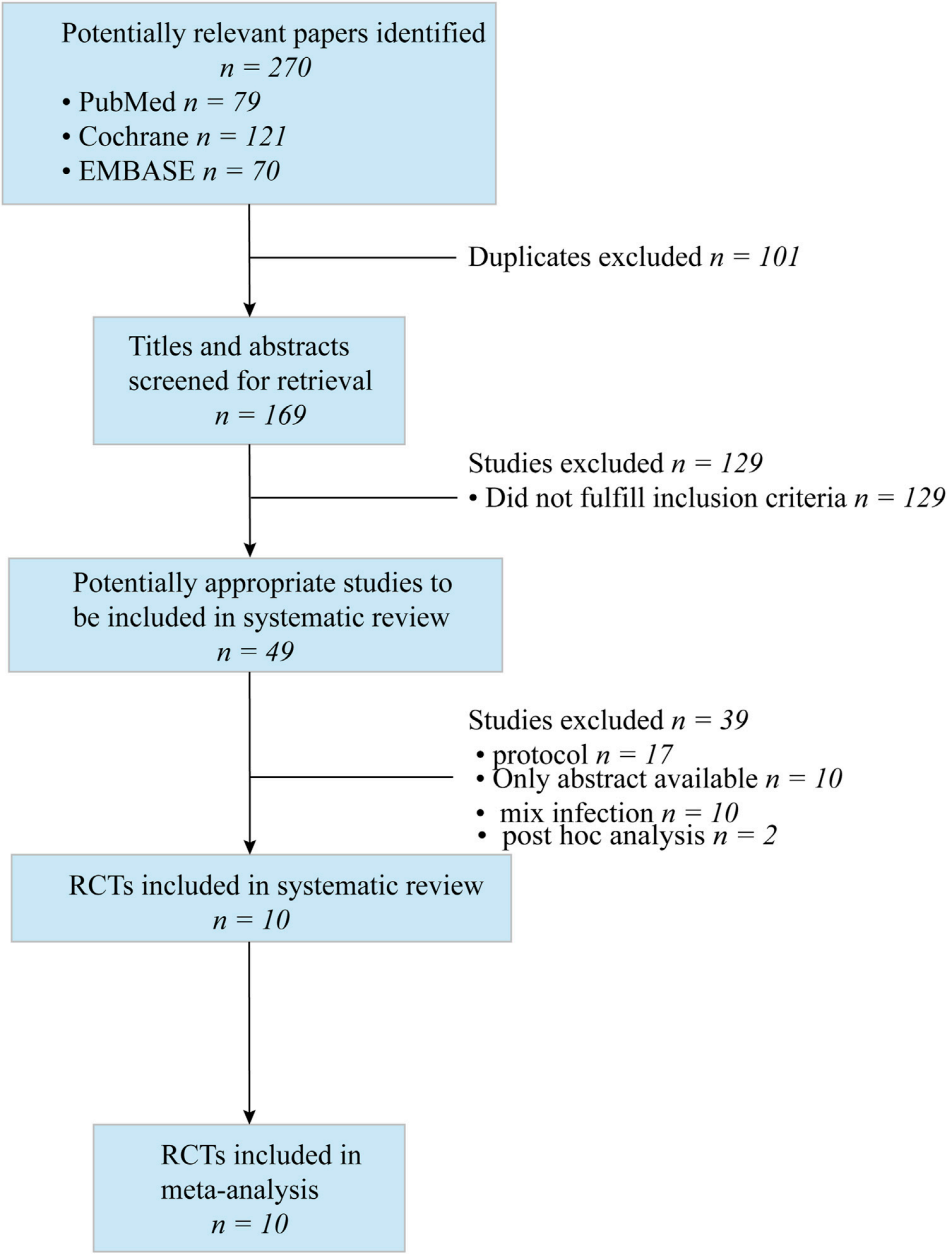
Flow dagram of literature search and study selection.

We searched CENTRAL, PubMed, and Embase, and identified 270 references. After primary screening and removal of duplicates, we excluded 101 articles. The full text of 49 articles was analyzed. Seventeen articles were protocols; 10 were conference abstracts; 10 reported mixed infections; and two articles reported *post hoc* analysis. A flow diagram of the literature search strategy and study selection is presented in Figure 1.

### 3.2 Characteristics of included studies and risk of bias

In the included studies, a total of 2,995 patients with cUTIs were treated with novel β-lactam antibiotics, while 2,930 patients were treated with other antibiotics. Ten RCTS were included in this systematic review. The main characteristics of the included studies are shown in Table 1. The novel β-lactam antibiotics investigated in the included studies were ceftazidime/avibactam (Vazquez et al., 2012; Wagenlehner et al., 2016; Carmeli et al., 2016), ceftolozane/tazobactam (Wagenlehner et al., 2015), imipenem-cilastatin plus relebactam (Sims et al., 2017), meropenem/vaborbactam (Kaye et al., 2018), cefiderocol (Portsmouth et al., 2018), cefepime/enmetazobactam (Kaye et al., 2022), sulopenem (Dunne et al., 2023), and tebipenem pivoxil hydrobromide (Eckburg et al., 2022). Drugs administered to the control group were imipenem_cilastatin, levofloxacin, doripenem, best-available therapy, piperacillin/tazobactam, and ertapenem. All studies reported the clinical response, microbiologic response, and AEs. Eight studies reported clinical cure rates at EOT and 6 studies reported SAEs, Five studies reported ACM. Microbiology of included studies are shown in Table 2. Five of the 10 studies permitted switch-to-oral treatment. Six studies reported patient populations based on the proportion of acute pyelonephritis (AP) or acute uncomplicated pyelonephritis (AUP) were more than to 50%, except to the study of Carmeli et al. (the best therapy group), Portsmouth et al.*,* Kaye, Eckburg et al. (ertapenem group) (Vazquez et al., 2012; Wagenlehner et al., 2016; Wagenlehner et al., 2015; Kaye et al., 2018; Portsmouth et al., 2018; Dunne et al., 2023). Eight studies reported female more than to 50%, except to the study of Carmeli et al., Sims et al. (Imipenem__cilastatin group) (Vazquez et al., 2012; Wagenlehner et al., 2016; Wagenlehner et al., 2015; Kaye et al., 2018; Portsmouth et al., 2018; Kaye et al., 2022; Dunne et al., 2023; Eckburg et al., 2022). Six studies indicated that the patients’ ages were below 60 years (Vazquez et al., 2012; Wagenlehner et al., 2016; Wagenlehner et al., 2015; Kaye et al., 2018; Kaye et al., 2022; Dunne et al., 2023). *Escherichia coli* was the predominant pathogen at baseline in ten studies.

**TABLE 1:** Characteristics of included studies.

Study	Number of participants	Population characteristics		Experimental group			Control group		
		Age (year) mean (SD)	Female/male (%)	Sample size	Intervention	Dose	Sample size	Intervention	Dose
Vazquez et al. (2012)	95	46.4 vs. 48.2	75 vs. 73	46	Ceftazidime/avibactam	2.5 g q8h	49	Imipenem_cilastatin	0.5 g q6h
Wagenlehner et al. (2015)	800	49.1 vs. 48.1	73.6 vs. 74.4	398	Ceftolozane/tazobactam	1.5 g q8h	402	Levofloxacin	0.75 g qd
Wagenlehner et al. (2016)	810	51.4 vs. 53.3	69.2 vs. 70.3	393	Ceftazidime/avibactam	2.5 g q8h	417	Doripenem	0.5 g q8h
Carmeli et al. (2016)	281	64.3 vs. 61.3	44 vs. 46	144	Ceftazidime/avibactam	2.5 g q8h	137	Best-available therapy	
Sims et al. (2017)	237	58–60 vs. 61	55 vs. 45	81	Imipenem-cilastatin plus relebactam	125 mg/250 mg	156	Imipenem__cilastatin	0.5 g q6h
Kaye et al. (2018)	545	53 vs. 52.3	66.5 vs. 65.9	272	Meropenem/vaborbactam	4 g q8h	273	Piperacillin__tazobactam	4.5 g q8h
Portsmouth et al. (2018)	371	62.3 vs. 61.3	53 vs. 60	252	Cefiderocol	2 g q8h	119	Imipenem__cilastatin	1 g q8h
Kaye et al. (2022)	1,034	55 vs. 54.3	54.7 vs. 55.2	516	Cefepime/enmetazobactam	2.5 g q8h	518	Piperacillin__tazobactam	4.5 g q8h
Dunne et al. (2023)	844	57.4 vs. 59.5	60.8 vs. 57	444	Sulopenem	1 g qd	440	ertapenem	1 g qd
Eckburg et al. (2022)	868	57.6 ± 18.7 vs. 58.7 ± 17.9	56.1 vs. 60.4	449	Tebipenem Pivoxil Hydrobromide	0.6 g q8h	419	ertapenem	1 g qd

**TABLE 2:** Microbiology of included studies.

Study	*Escherichia*. Coli (*E. coli*)	*Klebsiella pneumoniae*	*Enterobacter cloacae* (*E. cloacae*)	*Proteus* mirabillis (P.mirabilis)	*Pseudomonas* aenruginosa (P.aeruginosa)	*Citrobacter*. Koseri (C.koseri) (%)
Vazquez et al. (2012)	92.6% vs. 94.3%		0% vs. 2.9	0% vs. 2.9	7.4% vs. 0	3.7% vs. 0
Wagenlehner et al. (2015)	59.5% vs. 54.9%	5.3% vs. 3.4		0.5% vs. 1.7	1.5% vs. 1.7	
Wagenlehner et al. (2016)	74.3% vs. 73.4%	11.2% vs. 13.4	2.8% vs. 3.1	4.3% vs. 3.1	4.6% vs. 4.8	
Carmeli et al. (2016)	41% vs. 42%	38% vs. 47	6% vs. 4		14% vs. 5	
Sims et al. (2017)	64.8% vs. 63.3%	11.3% vs. 13.9			7.0% vs. 7.6	
Portsmouth et al. (2018)	60.3% vs. 66.4%	19 %vs. 21	3.6% vs. 0.8	6.7% vs. 1.7	7.1% vs. 4.2	
Kaye et al. (2018)	65.1% vs. 64.3%	15.6% vs. 15.4	5.2% vs. 2.7	3.1% vs. 6.6		
Kaye et al. (2022)	76.4%	9.7	1.5	5.6	3.5	
Dunne et al. (2023)	76.1% vs. 78.6%	12.6% vs. 10.7				
Eckburg et al. (2022)	>90% Enterobacterales					

**FIGURE 2:**
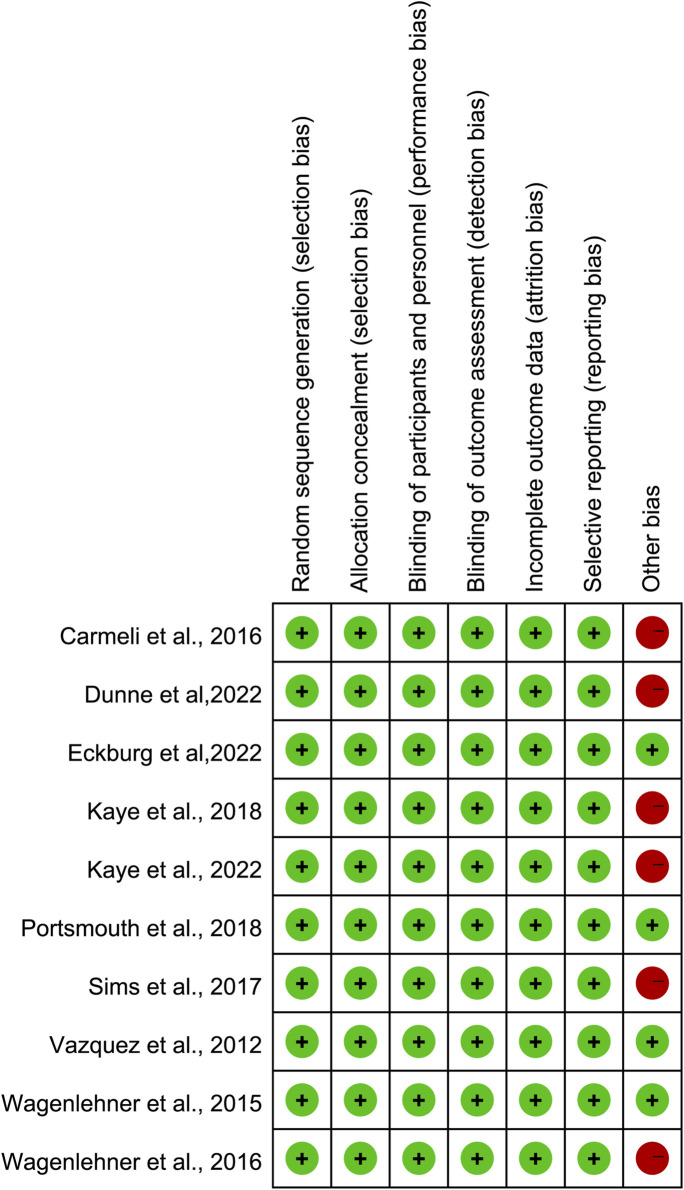
Assessment of the risk of bias.

The Cochrane Risk of Bias 2.0 assessment tool was used to evaluate the quality of the included studies. Ten studies had a very low risk of sequence generation, performance bias, attrition bias, and reporting bias. Six studies supported by a commercial company carried a high risk of other bias (Wagenlehner et al., 2016; Carmeli et al., 2016; Sims et al., 2017; Kaye et al., 2018; Kaye et al., 2022; Dunne et al., 2023; Eckburg et al., 2022). The outcomes of the risk of bias assessment are summarized in Figure 2.

### 3.3 Clinical response

#### 3.3.1 Overall clinical response

**FIGURE 3:**
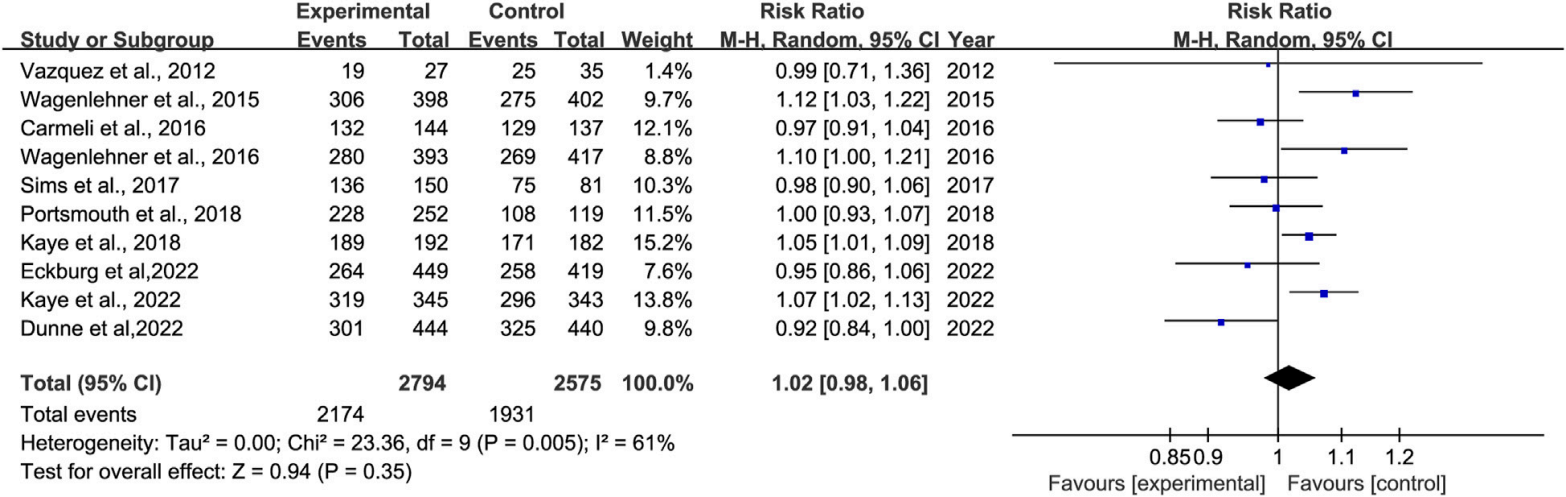
Overall clinical response between other antibiotics groups versus novel β-lactam antibiotics groups for the treatment of cUTIs.

Overall Clinical response was reported in 10 studies (Vazquez et al., 2012; Wagenlehner et al., 2016; Carmeli et al., 2016; Wagenlehner et al., 2015; Sims et al., 2017; Kaye et al., 2018; Portsmouth et al., 2018; Kaye et al., 2022; Dunne et al., 2023; Eckburg et al., 2022). There was no significant difference in overall clinical response, with moderate heterogeneity between other antibiotics groups versus novel β-lactam antibiotics groups (RR = 1.01, 95% CI 0.98–1.06, I^2^ = 61%, P = 0.35, Figure 3).

#### 3.3.2 Clinical cure rates at TOC

**FIGURE 4:**
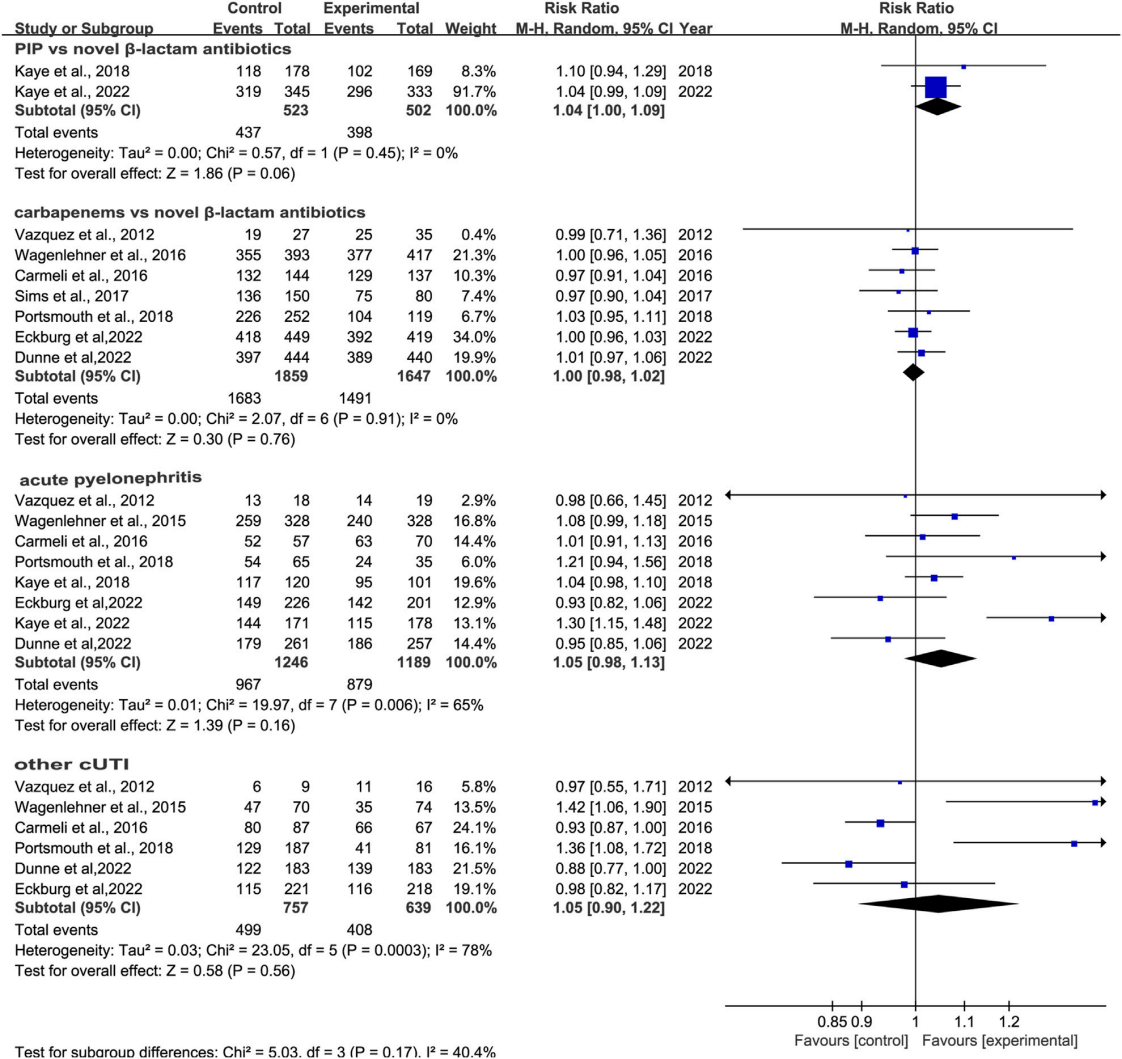
Clinical cure rates at TOC between novel β-lactam antibiotics groups versus carbapenems groups for the treatment of cUTIs.

We conducted subgroup analysis of differences in clinical cure rates at TOC between novel β-lactam antibiotics groups and carbapenems groups. Results revealed that there was no difference, with low heterogeneity (RR = 1.0, 95% CI 0.98–1.02, I^2^ = 0%, P = 0.76, Figure 4). Subgroup analysis of the difference in clinical cure rates at TOC between novel β-lactam antibiotics groups and piperacillin/tazobactam groups (RR = 1.04, 95% CI 1.00–1.09, I^2^ = 0%, P = 0.06, Figure 4) revealed that there was no difference. We conducted a subgroup analysis of the difference in clinical cure rates at TOC for acute pyelonephritis between novel BBL/BBLs groups and other antibiotics groups (RR = 1.05, 95% CI 0.98–1.13, I^2^ = 65%, P = 0.16, Figure 4) and there was no difference. A subgroup analysis of the difference in clinical cure rates at TOC for other cUTIs between novel BBL/BBLs groups and other antibiotics groups also revealed no difference (RR = 1.05, 95% CI 0.90–1.22, I^2^ = 7 8%, P = 0.56, Figure 4). We conducted a sensitivity analysis of clinical cure rates at TOC, and the results were stable (Table 3).

**TABLE 3:** Sensitivity analyse (omission of a single RCT)*.

	TOC(95%CI)	P
All studies	1.01 (−0.99–1.04)	0.26
selected study omitted		
Vazquez et al. (2012)	1.01 (0.99–1.04)	0.24
Wagenlehner et al. (2015)	0.01 (0.99–1.03)	0.5
Wagenlehner et al. (2016)	1.02 (0.99–1.05)	0.21
Carmeli et al. (2016)	1.02 (0.99–1.04)	0.14
Sims et al. (2017)	1.02 (0.99–1.04)	0.15
Portsmouth et al. (2018)	1.01 (0.99–1.03)	0.28
Kaye et al. (2018)	1.01 (1.01–1.03)	0.36
Kaye et al. (2022)	1.01 (0.98–1.04)	0.50
Dunne et al. (2023)	1.01 (0.99–0.04)	0.28
Eckburg et al. (2022)	1.02 (0.99–1.04)	0.16

#### 3.3.3 Clinical cure rates at EOT

**FIGURE 5:**
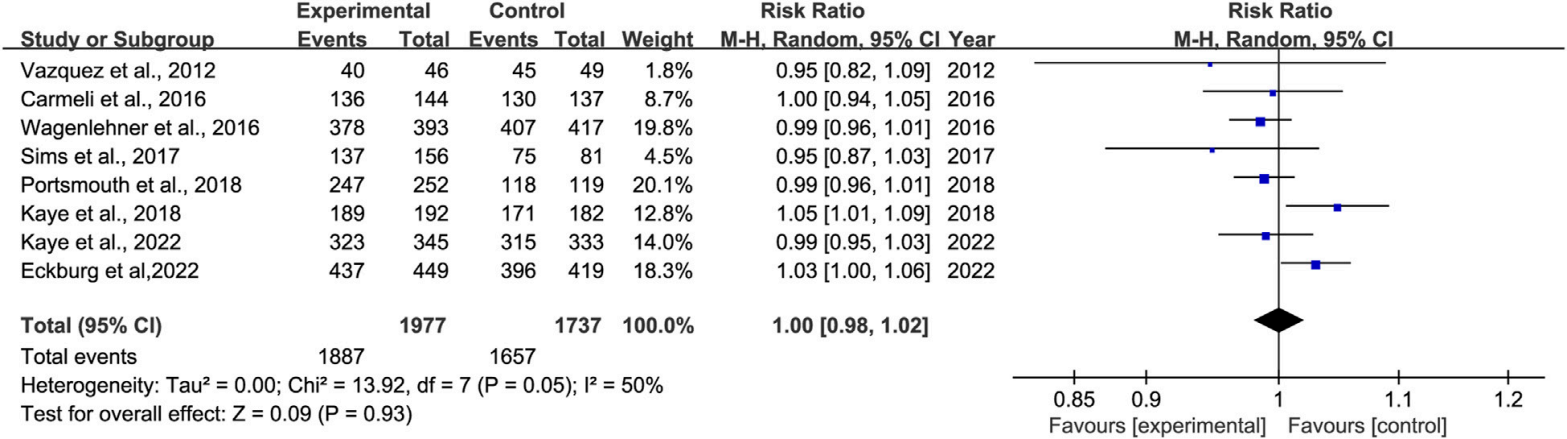
Clinical cure rates at EOT between novel β-lactam antibiotics groups versus other antibiotics groups for the treatment of cUTIs.

**FIGURE 6:**
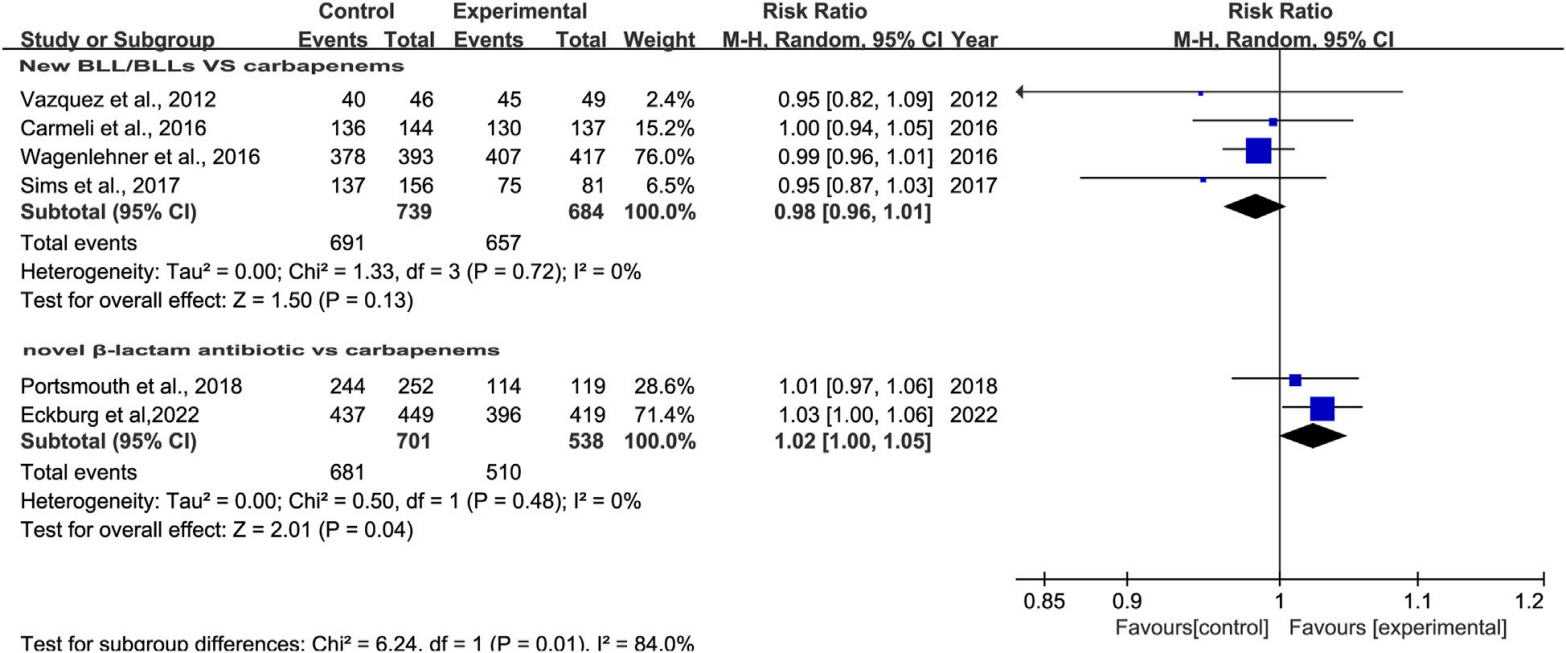
Subgroup analysis of clinical cure rates at EOT between novel β-lactam antibiotics groups versus carbapenems groups for the treatment of cUTIs.

Eight studies reported clinical cure rates at EOT. Subgroup analysis of the difference in clinical cure rates at EOT between novel β-lactam antibiotics groups and other antibiotics groups revealed that there was no difference, with moderate heterogeneity (RR = 1.0, 95% CI 0.98–1.02, I^2^ = 50%, P = 0.93, Figure 5). Subgroup analysis of BLL/BLS groups versus carbapenems groups indicated no difference, with low heterogeneity (RR = 0.98, 95% CI 0.96–1.01, I^2^ = 0%, P = 0.13, Figure 6). Furthermore, we conducted a subgroup analysis of clinical cure rates at EOT between novel β-lactam antibiotics groups versus carbapenems groups and found significant differences, with low heterogeneity (RR = 1.02, 95% CI 1.0–1.05, I^2^ = 0%, P = 0.04, Figure 6).

### 3.4 Microbiological response

**FIGURE 7:**
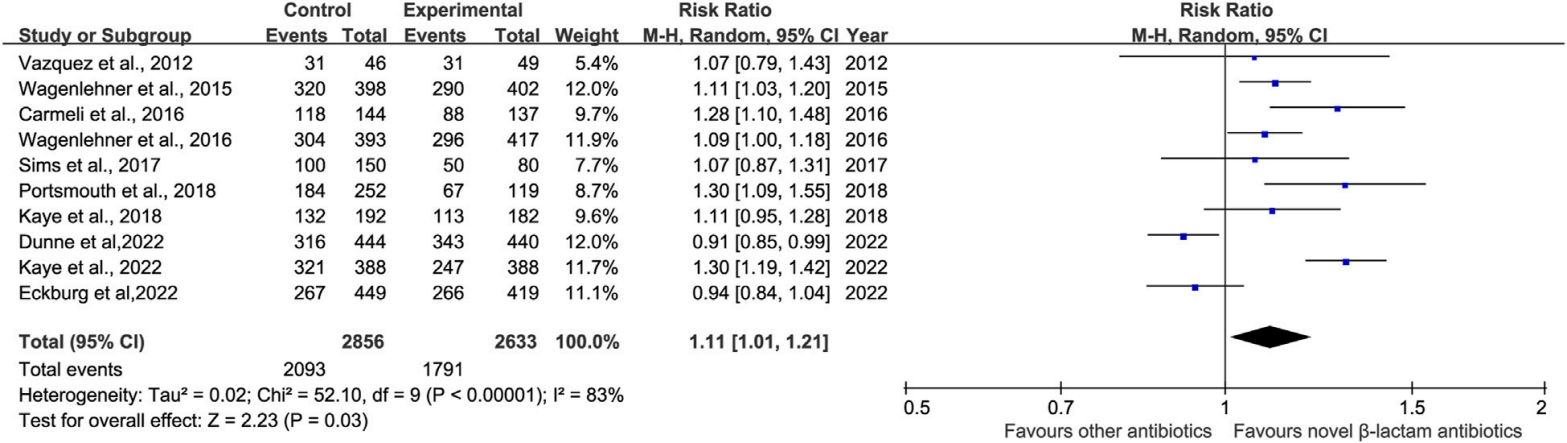
Microbiological response between novel β-lactam antibiotics groups versus other antibiotics groups for the treatment of cUTIs.

**FIGURE 8:**
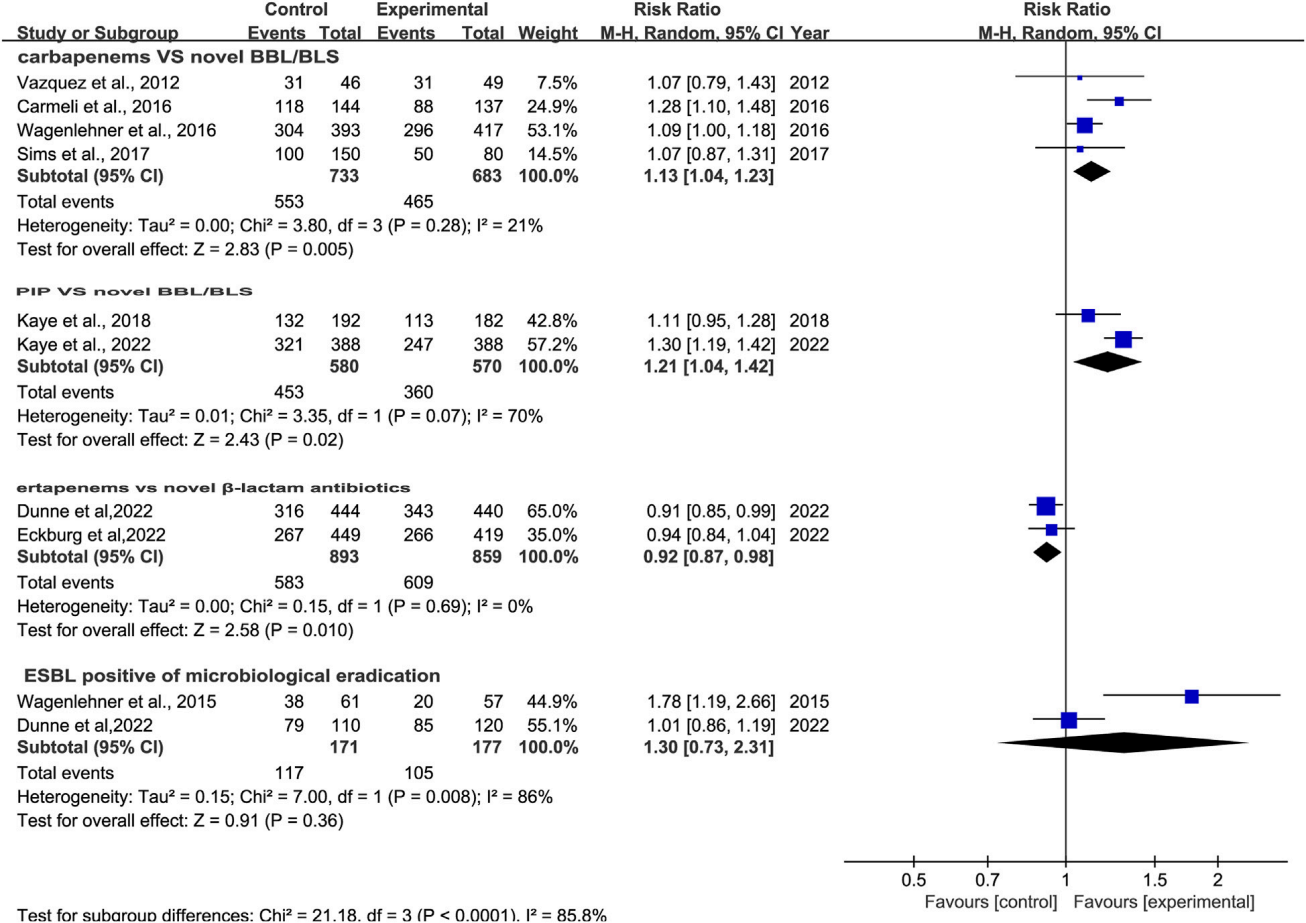
Subgroup analysis of microbiological response between novel β-lactam antibiotics groups versus other antibiotics groups for the treatment of cUTIs.

Microbiological response was reported in ten studies (Vazquez et al., 2012; Wagenlehner et al., 2016; Carmeli et al., 2016; Wagenlehner et al., 2015; Sims et al., 2017; Kaye et al., 2018; Portsmouth et al., 2018; Kaye et al., 2022; Dunne et al., 2023; Eckburg et al., 2022). There was a difference in microbiological response between novel β-lactam antibiotics groups and other antibiotics groups, with substantial heterogeneity (RR = 1.11, 95% CI 1.01–1.21, I^2^ = 83%, P = 0.03, Figure 7). To explore the heterogeneity, we conducted subgroup analysis of differences in microbiological eradication between novel β-lactam and β-lactamase inhibitors groups versus carbapenems groups; results revealed that there was a significant difference, with low heterogeneity (RR = 1.13, 95% CI 1.04–1.23, I^2^ = 21%, P = 0.005, Figure 8). Subgroup analysis of differences between novel BBL/BBLs groups and piperacillin/tazobactam sodium groups revealed that there was a difference, with substantial heterogeneity (RR = 1.21, 95% CI 1.04–1.42, I^2^ = 70%, P = 0.02, Figure 8). Subgroup analysis of differences in microbiological eradication between novel β-lactam antibiotics groups and ertapenem groups revealed a significant difference (RR = 0.92, 95% CI 0.87–0.98, I^2^ = 0, P = 0.01, Figure 8). The type of antibiotics was the source of the heterogeneity. Subgroup analysis of microbiological eradication of ESBL-positive bacteria revealed no difference between novel β-lactam antibiotics groups and other antibiotics groups (RR = 1.3, 95% CI 0.73–2.31, I^2^ = 86%, P = 0.36, Figure 8).

### 3.5 Side-effects of antibiotic treatment

#### 3.5.1 Adverse effects

**FIGURE 9:**
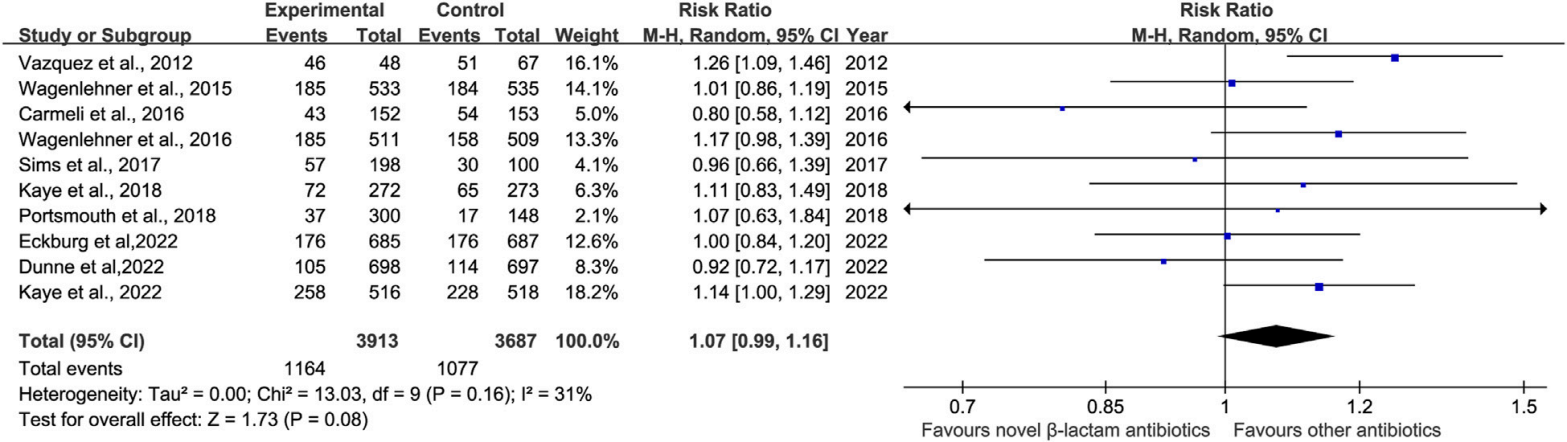
AEs between novel β-lactam antibiotics groups versus other antibiotics groups for the treatment of cUTIs.

**FIGURE 10:**
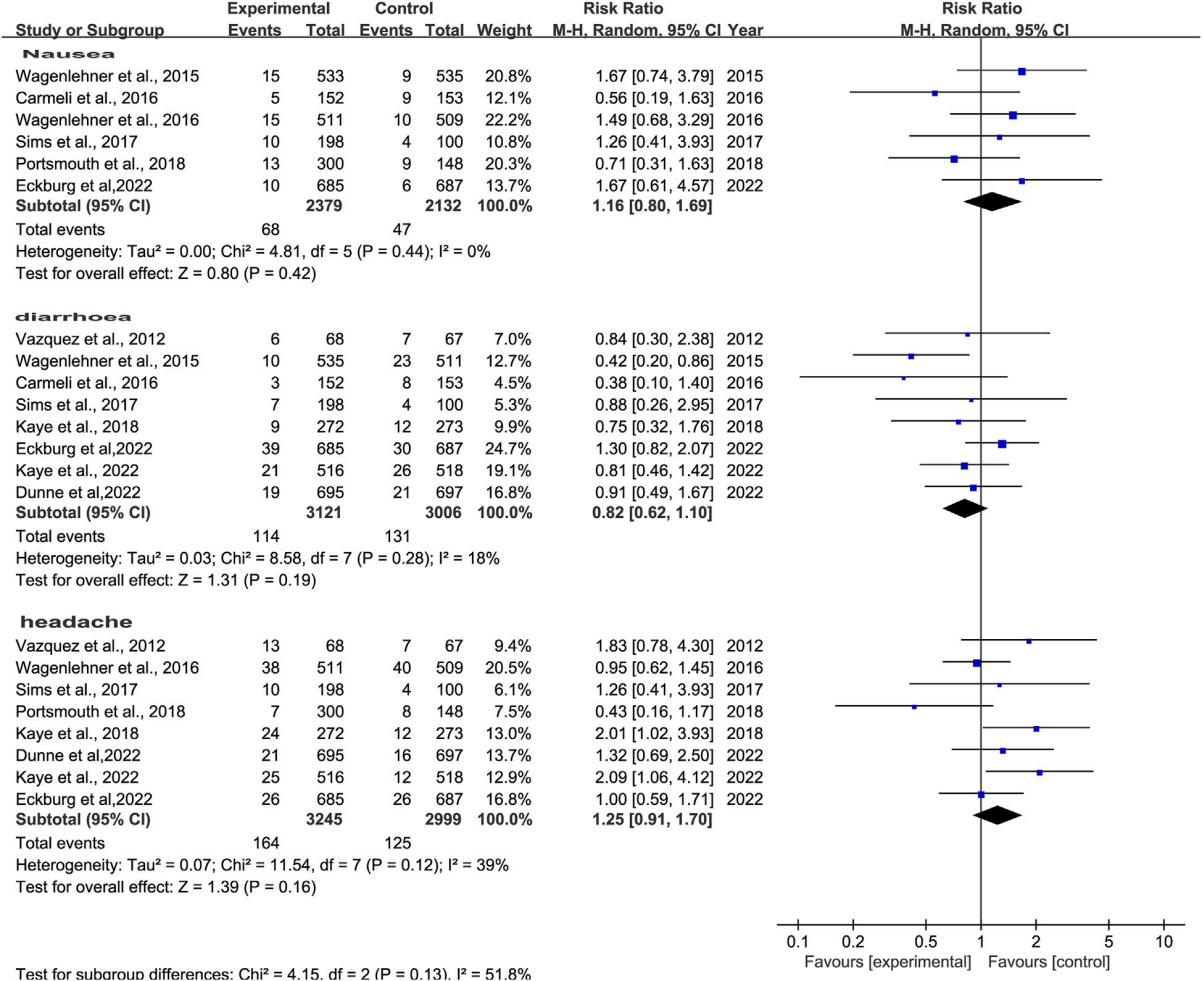
Subgroup analysis of common AEs between other antibiotics groups versus novel β-lactam antibiotics groups for the treatment of cUTIs.

The prevalence of adverse effects (AEs) was reported in 10 studies (Vazquez et al., 2012; Wagenlehner et al., 2016; Carmeli et al., 2016; Wagenlehner et al., 2015; Sims et al., 2017; Kaye et al., 2018; Portsmouth et al., 2018; Kaye et al., 2022; Dunne et al., 2023; Eckburg et al., 2022). There was no significant difference in AEs with low heterogeneity between novel β-lactam antibiotics groups versus other antibiotics groups (RR = 1.07, 95% CI 0.99–1.16, I^2^ = 31%, P = 0.08, Figure 9). We also conducted subgroup analysis of common AEs: no significant difference was observed between other antibiotics groups and novel β-lactam antibiotics groups in terms of nausea (RR = 1.16, 95% CI 0.80–1.69, I^2^ = 0%, P = 0.42, Figure 10), diarrhea (RR = 0.82, 95% CI 0.62–1.10, I^2^ = 18%, P = 0.19, Figure 10), and headache (RR = 1.25, 95% CI0.91–1.70, I^2^ = 39%, P = 0.16, Figure 10).

#### 3.5.2 Serious adverse effects

**FIGURE 11:**
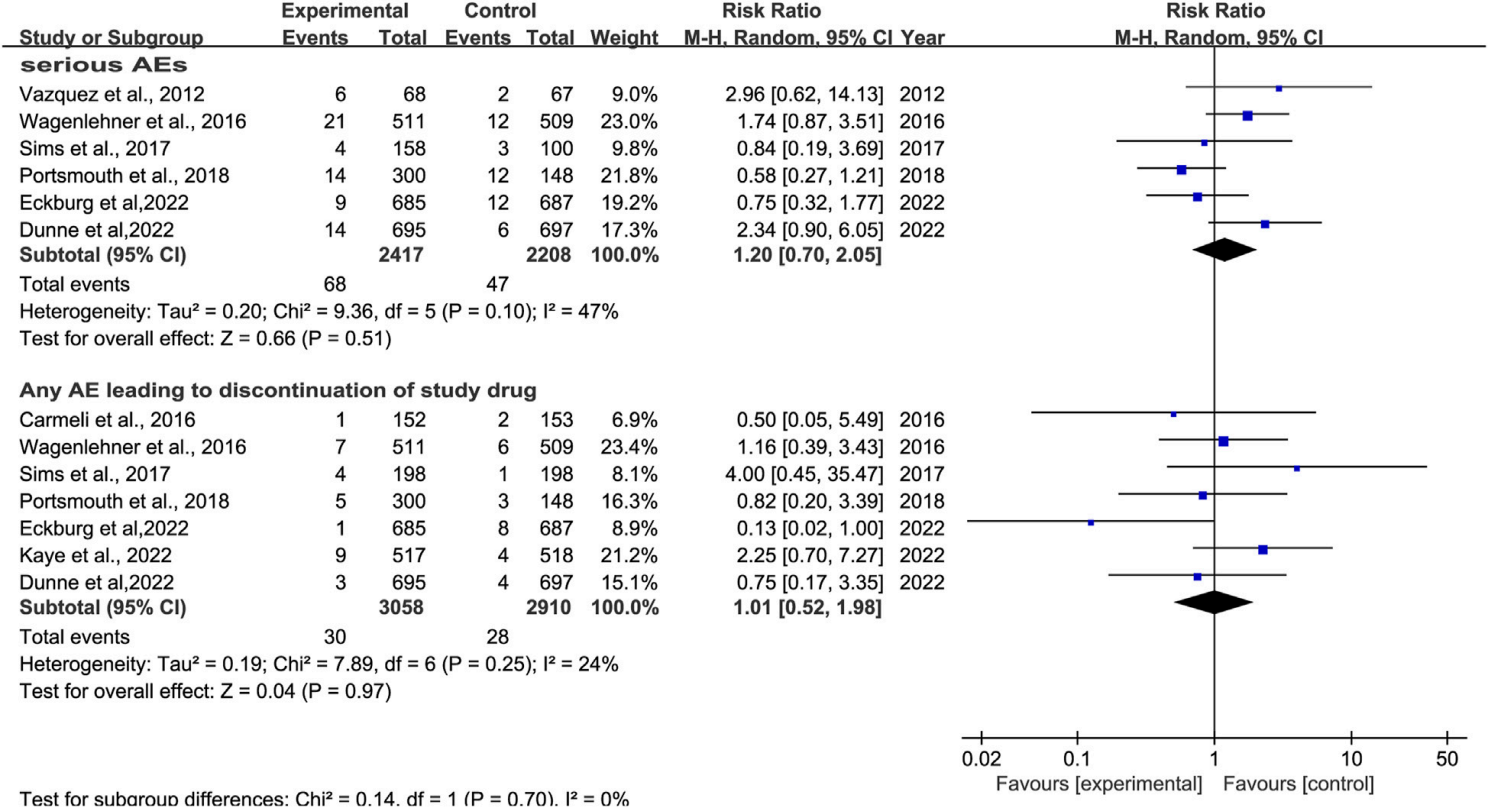
SAEs between novel β-lactam antibiotics groups versus other antibiotics groups for the treatment of cUTIs.

Six studies reported serious AEs (Vazquez et al., 2012; Wagenlehner et al., 2016; Carmeli et al., 2016; Portsmouth et al., 2018; Dunne et al., 2023; Eckburg et al., 2022). There was no significant difference in serious AEs, with low heterogeneity, between novel β-lactam antibiotics groups versus other antibiotics groups (RR = 1.20, 95% CI 0.70–2.05, I^2^ = 47%, P = 0.51, Figure 11). Six studies reported events that led to study discontinuation. We conducted subgroup analysis and found no difference, with low heterogeneity, between novel β-lactam antibiotics and other antibiotics (RR = 1.01, 95% CI 0.52–1.98, I^2^ = 24%, P = 0.97, Figure 11).

### 3.6 Publication bias

**FIGURE 12:**
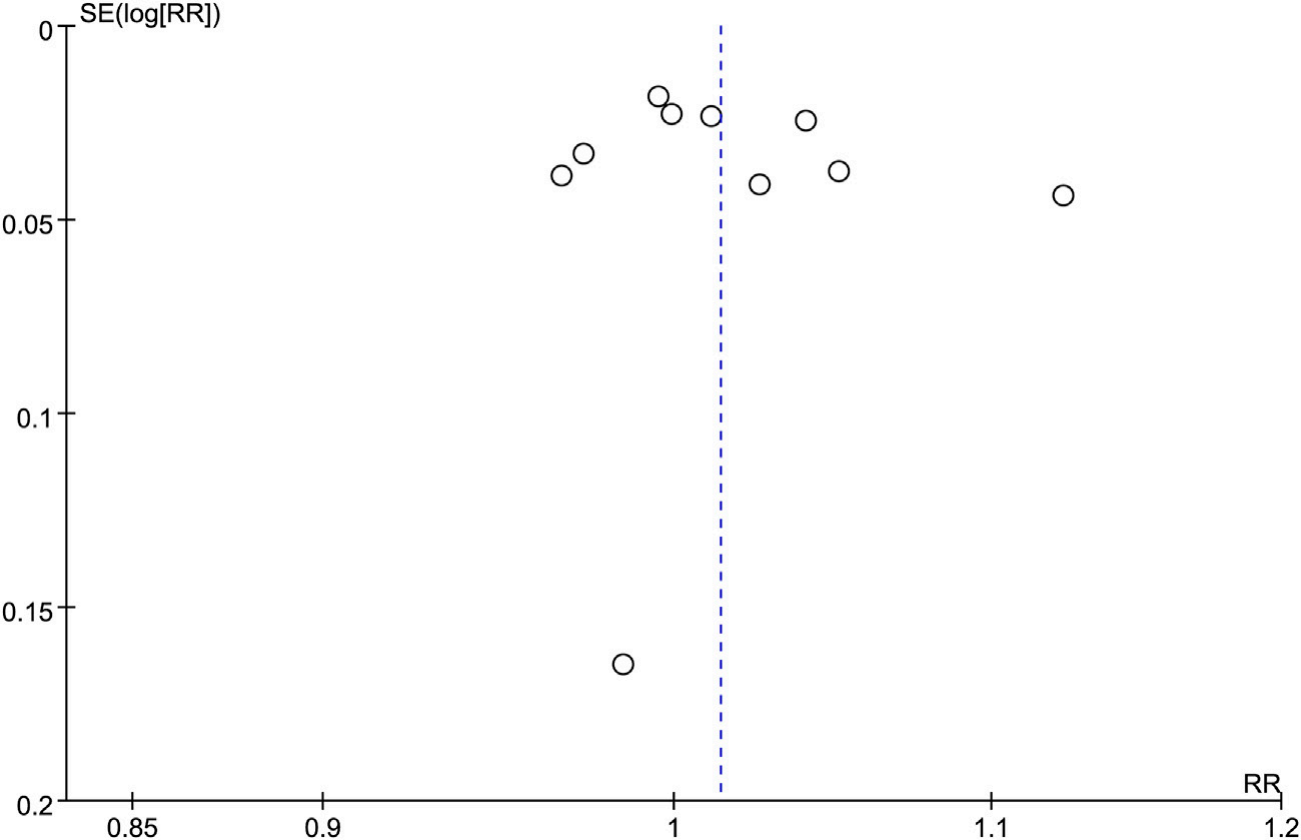
Funnel plot for clinical response at TOC.

Publication bias was evaluated using a funnel plot, and found to be low for clinical cure (Figure 12), microbiological response, and AEs.

## 4 Discussion

In this meta-analysis, we found no significant difference in the overall clinical response between patients with cUTIs treated with novel β-lactam antibiotics versus other antibiotics. Similar results were observed concerning AEs and SAEs. However, we found that patients treated with novel β-lactam antibiotics had higher clinical cure rates at EOT in a subgroup analysis than patients who were treated with carbapenems. We found novel β-lactam antibiotics to be superior to other antibiotics in regard to microbiologic response. We also observed that novel BBL/BBLs are superior to carbapenems and piperacillin/tazobactam sodium in regard to microbiologic response. This meta-analysis included 10 RCTs in which novel β-lactam antibiotics, including ceftazidime/avibactam, ceftolozane/tazobactam, cefepime/enmetazobactam, imipenem/cilastatin plus relebactam, meropenem/vaborbactam, cefiderocol, sulopenem, tebipenem pivoxil hydrobromide were used for the treatment of patients with cUTIs. We conducted sensitivity analysis of microbiological response and found the result to be stable. Furthermore, we conducted subgroup analysis according to antibiotic type and infection type in terms of clinical response, and found no difference between novel β-lactam antibiotics and other antibiotics.

Although extant systematic reviews and meta-analyses have compared novel antibiotics with other antibiotics in terms of their efficacy and safety for the treatment of cUTIs, comparisons with novel β-lactam antibiotics have not been reported to date. Subgroup analysis according to the specific type of novel antibiotics and infection type in terms of clinical response has yet to be performed. Ezure et al. examined only two types of novel β-lactam antibiotics, and only in regard to clinical response to TOC, with substantial heterogeneity; furthermore, their study did not include analysis of clinical response at EOT (Ezure et al., 2022). In comparison, our study included eight types of novel β-lactam antibiotics. Moreover, in contrast to a previous study (Hung et al., 2023), we found novel β-lactam antibiotics to be similar to other antibiotics in terms of clinical response at TOC, with low heterogeneity; however, subgroup analysis of clinical cure rates at EOT revealed significant differences between novel β-lactam antibiotics and carbapenems.

The concern of antimicrobial resistance (AMR) is considered to be one of the top ten threats to global health. Several factors are recognized as relevant etiological contributors to the emergence of AMR, including the worldwide misuse or overuse of antibiotics. The emergence of carbapenem-resistant *K. pneumoniae* (CRKP) isolates poses a significant public health threat. The identification of a diverse array of resistance mechanisms in *Pseudomonas aeruginosa* has become a major concern for healthcare institutions worldwide, particularly in the context of invasive therapeutic procedures. The treatment of *P. aeruginosa* infections is increasingly challenging for clinicians due to the limited range of available therapies. As a result of both acquired and intrinsic resistance mechanisms, multi-drug resistant (MDR) strains of this species are now commonplace in clinical practice (Algammal et al., 2023; Karampatakis et al., 2023; Behzadi et al., 2022). The appropriate choice of antibiotics is critical for treatment of cUTIs, which often involve MDR bacteria. A previous study reported that other antibiotics such as cephalosporin (42%) and ciprofloxacin (54.9%) were associated with a much higher overall resistance rate. Therefore, the options for adequate empiric antibiotic therapy in cUTIs are limited (Choe et al., 2018). To avoid exacerbating antimicrobial resistance, carbapenems should not be overused. This systematic review found that novel β-lactam antibiotics may be an appropriate choice for treating patients with cUTIs. Novel β-lactam antibiotics were found to be superior to carbapenems in terms of clinical cure rates at EOT and elicited a better microbiological response than other antibiotics. The microbiological response to novel β-lactam antibiotics has been explained in numerous previous *in vitro* studies. Ceftazidime/avibactam is a novel BBL/BBL that inhibits Ambler class A, Class C, and some class D β-lactamases *in vitro* and restores the antimicrobial activity of ceftazidime against certain MDR pathogens (Stone et al., 2018). Karlowsky et al. demonstrated that ceftolozane-tazobactam inhibited 95.6% of all isolates of *E. coli* and 90.0% of ESBL-positive, carbapenemase-negative *E. coli* at its susceptible MIC breakpoint (Karlowsky et al., 2020). The susceptibility of *Enterobacterales* to cefiderocol was 99.8%; that of carbapenem-resistant Enterobacterales (CRE) was 98.2%; and 99.6% of *P. aeruginosa* isolates in the US and Europe were found to susceptible (Shortridge et al., 2022). Karlowsky et al. reported that imipenem/cilastatin plus relebactam inhibited 71% of MDR *P. aeruginosa* isolates, while relebactam restored imipenem susceptibility to 70% of imipenem-non susceptible isolates and 94.8% of resistant *K. pneumoniae* isolates (Karlowsky et al., 2018a; Karlowsky et al., 2018b). The study found that the activity of meropenem-vaborbactam (MIC50/90, 0.06/2 mg.L^−1^) was superior to that of meropenem, while other β-lactam agents were found to inhibit CRE isolates in the US (Castanheira et al., 2020). Cefepime-enmetazobactam inhibited 99.7% of all *E. coli* isolates and 93.2% of *K. pneumoniae* at the breakpoint of 1 μg mL^−1^ (Morrissey et al., 2019). Sulopenem was shown to inhibit 99.2% of Enterobacterales isolates at ≦1 mg. L^−1^ (Maher et al., 2023). Arends SJR et al. observed that the MIC90 values of tebipenem were ≤0.12 mg.L^−1^—eight folds greater than those of imipenem against *E. coli* and *K. pneumoniae* (Arends et al., 2019). Although piperacillin/tazobactam is an alternative option for infections caused by ESBL-positive pathogens, a recent study showed that piperacillin/tazobactam was inferior to meropenem when used for treatment of patients with infections with ESBL-positive pathogens (Harris et al., 2018). The study found only 50% of the isolated UPEC strains exhibited sensitivity of imipenem, while resistance to imipenem was observed in 34% of the isolated UPEC pathotypes (Khonsari et al., 2021).

The previous studies demonstrate the superior effectiveness of novel β-lactam antibiotics over carbapenems for the treatment of patients with cUTIs (Vazquez et al., 2012; Wagenlehner et al., 2016; Carmeli et al., 2016; Sims et al., 2017; Kaye et al., 2018; Portsmouth et al., 2018). Potsmouths S et al. reported cefiderocol to be superior to imipenem-cilastatin in *post hoc* analysis (Portsmouth et al., 2018). Several studies have reported that novel β-lactam antibiotics demonstrate superior efficacy against multidrug-resistant infections. A multicenter cohort study identified ceftolozane-tazobactam and ceftazidime-avibactam as promising therapeutic options for the management of infections caused by multidrug-resistant *Pseudomonas aeruginosa* (Almangour et al., 2023). Bassetti et al. reported that cefiderocol demonstrated comparable clinical and microbiological efficacy to the best available therapy in patients with infections caused by carbapenem-resistant Gram-negative bacteria, thereby supporting its use as a viable treatment option for carbapenem-resistant infections in individuals with limited therapeutic alternatives (Bassetti et al., 2021). Clinical success was observed for both ceftazidime-avibactam and meropenem/vaborbactam in the treatment of KPC-producing CRE infections (Ackley et al., 2020). Overall, novel β-lactam antibiotics showed better activity against gram-negative bacterium, including CRE. In regard to safety, we found that the prevalence of common AEs, including nausea, diarrhea, and headache, were similar to those for other antibiotics. Similar results were observed in regard to SAEs.

This meta-analysis and systematic review had some limitations. First, all included studies did not report clinical responses according to disease severity; as a result, relevant subgroup analysis could not be conducted. Second, all studies included were RCTs pertaining to regulatory and marketing approval, which do not reflect “real-world” treatment outcomes. Furthermore, none of the studies provided data on the inhibition of MDR organisms or CRE, thereby failing to highlight the advantages of novel agents capable of treating MDR pathogens. Third, Sulopenem and tebipenem were not granted approval by the FDA or EMA. Regarding the primary endpoint, the noninferiority of sulopenem compared to ertapenem was not established. This discrepancy was primarily attributed to a reduced incidence of asymptomatic bacteriuria in the subgroup of patients treated with ertapenem who subsequently transitioned to ciprofloxacin (Dunne et al., 2023). Given the ongoing rise in antibacterial resistance, it is imperative to critically evaluate the appropriateness of fluoroquinolones as comparators (Portsmouth et al., 2021). Finally, only one study reported colitis due to *Clostridium difficile*, which also limited the available data and prevented us from performing subgroup analysis.

## 5 Conclusion

Novel β-lactam antibiotics, in a subgroup analysis, showed a superior clinical response in EOT compared with carbapenems. Moreover, novel β-lactam antibiotics also elicited a stronger microbiologic response than other antibiotics. The study population predominantly consisted of individuals with AP, with most studies including a higher proportion of younger patients and females. *Escherichia coli* emerged as the predominant pathogen, leading to enhanced clinical and microbiological response rates. The safety of novel β-lactam antibiotics was similar to that of other antibiotics. Carbapenemase-producing *E. coli* infections have become a global public health threat and are associated with high morbidity and mortality (Boutzoukas et al., 2023). The identification of alternative effective antibiotics is therefore critical. Novel β-lactam antibiotics with the extended activity; these encouraging results support the use of novel β-lactam antibiotics as a potential alternative to carbapenems in patients with carbapenem-resistant gram-negative infections. Larger sample sizes comprising patients from different ethnic populations are required to confirm our findings.

## Data Availability

The original contributions presented in the study are included in the article/Supplementary Material, further inquiries can be directed to the corresponding author.
