# Supplementary material for: Novel β-lactam antibiotics versus other antibiotics for treatment of complicated urinary tract infections: a systematic review and meta-analysis
Source: Front Pharmacol. 2024 Oct 10;15:1420170. doi: 10.3389/fphar.2024.1420170 (PMC11500039; doi:10.3389/fphar.2024.1420170)
Supplement: Supplementary file 1 [file Table1.docx]

Supplementary Material

# Supplementary Data

Search strategy

Database:Pubmed

| # | Searches |
| --- | --- |
| 1 | "Urinary Tract Infections"[Mesh] |
| 2 | "Urinary Tract Infections"[Mesh] |
| 3 | "Pyuria"[Mesh] |
| 4 | "Pyuria"[Mesh] |
| 5 | "Pyuria"[Mesh] |
| 6 | urinary tract infection* |
| 7 | UTI[Title/Abstract] OR UTIs[Title/Abstract] |
| 8 | bacteriuria[Title/Abstract] |
| 9 | pyuria[Title/Abstract] |
| 10 | pyelonephritis[Title/Abstract] |
| 11 | OR 1~10 |
| 12 | "ceftazidime/avibactam" OR "avycaz" OR "zavicefta" OR "ceftolozane/tazobactam"OR "zerbaxa" OR "meropenem/vaborbactam" OR "vabomere" OR "vaborem"OR "imipenem/cilastatin/relebactam" OR "imipenem/relebactam" OR "recarbrio"OR "cefepime/tazobactam" OR "aztreonam/avibactam" OR "ceftaroline/avibactam" OR "cefepime/zidebactam" OR"cefepime-enmetazobactam" OR "WCK 5222" OR "Meropenem/nacubactam" OR "Cefiderocol" or "Sulopenem" OR "Tebipenem Pivoxil Hydrobromide " |
| 13 | #11 AND#12 |
| 14 | (randomized controlled trial[pt] OR controlled clinical trial[pt] OR randomized[tiab] OR placebo[tiab] OR clinical trials as topic[mesh:noexp] OR randomly[tiab] OR trial[ti]) NOT (animals [mh] NOT (humans [mh] AND animals[mh])) |
| 15 | (randomized controlled trial[pt] OR controlled clinical trial[pt] OR randomized[tiab] OR placebo[tiab] OR clinical trials as topic[mesh:noexp] OR randomly[tiab] OR trial[ti]) NOT (animals [mh] NOT (humans [mh] AND animals[mh])) |

**
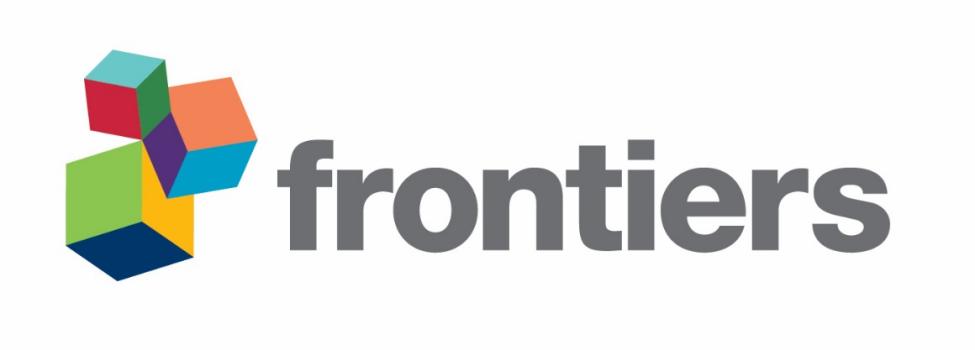
**
